# High-altitude hypoxia exacerbates gastric mucosal damage and regulates the Nrf2 signaling pathway in *Helicobacter pylori*-infected mice

**DOI:** 10.3389/fphys.2025.1724998

**Published:** 2026-01-09

**Authors:** Chunxia Li, Xuehong Wang, Sen Cui

**Affiliations:** 1 Department of Gastroenterology, Qinghai University Affiliated Hospital, Xining, Qinghai, China; 2 Clinical Medical College of Qinghai University, Xining, Qinghai, China; 3 Department of Hematology, Qinghai University Affiliated Hospital, Xining, Qinghai, China

**Keywords:** gastric mucosal barrier, *Helicobacter pylori*, hypoxia, Inflammation, Nrf2, oxidative stress

## Abstract

**Introduction:**

*Helicobacter pylori* (*H. pylori*) infection is a primary etiological factor in gastric mucosal injury. High-altitude hypoxic environments are suspected to exacerbate this damage, although the precise mechanisms remain poorly defined. This study aimed to investigate the impact of high-altitude hypoxia on the gastric mucosal barrier and the Nrf2 signaling pathway in *H. pylori*-infected mice.

**Methods:**

Male C57BL/6 mice were randomly divided into four groups: the control group (Con), the hypoxia group (H), the *H. pylori* infection group (Hp), and the combined *H. pylori* infection with hypoxia group (HpH), with 10 mice per group. A mouse model of *H. pylori* infection under hypoxic conditions was established by combining a hypobaric chamber simulating an altitude of 5000 m with *H. pylori* gavage. Pathological changes in the gastric mucosa were observed by HE staining. The expression of tight junction proteins, apoptosis-related proteins, oxidative stress markers, inflammatory factors, and key molecules of the Nrf2 pathway in gastric tissues were evaluated using qRT-PCR, immunohistochemistry, Western blot, and biochemical analysis.

**Results:**

Compared to the H and Hp groups, mice in the HpH group exhibited significantly higher gastric mucosal epithelial damage scores. This group also showed decreased expression of ZO-1, Occludin, and Bcl-2 in gastric tissues, along with increased expression of Bax and Caspase-3. Furthermore, in the HpH group, the gastric levels of MDA, TNF-α, IL-1β, and IL-6 were elevated, while the activities of SOD and GSH-Px were reduced. Additionally, the HpH group displayed increased expression levels of Keap1 in gastric tissues, along with decreased levels of Nrf2 and its downstream target genes HO-1 and NQO1.

**Discussion:**

High-altitude hypoxia exacerbates oxidative stress and inflammatory responses induced by *H. pylori* infection, reduces tight junction protein expression, and triggers changes in apoptosis-related protein expression, exacerbating the disruption of the gastric mucosal barrier, consequently leading to more severe gastric mucosal damage. The underlying mechanism may be associated with the inhibition of the Nrf2 signaling pathway in the gastric tissues.

## 1 Introduction

*H. pylori* (*H. pylori*) infection is the primary cause of chronic gastritis globally, leading to the development of chronic active gastritis in nearly all infected individuals (Rugge et al., 2021; Chinese Society of Gastroenterology, 2023). A recently published systematic review and meta-analysis indicates that the global *H. pylori* infection rate has declined from 58.2% in 1980–1990 to 43.1% in 2011–2022 (Li et al., 2023), yet it continues to impose a substantial clinical and public health burden. High-altitude environments are widely distributed across the globe, with an estimated 81.6 million people residing in high-altitude regions at an elevation of over 2,500 m (Tremblay and Ainslie, 2021). As altitude increases, atmospheric oxygen partial pressure decreases, reducing the amount of oxygen available to the body, and this hypobaric hypoxia is the primary factor affecting physiological functions in high-altitude environments (Luks and Hackett, 2022). Multiple studies have demonstrated that *H. pylori* infection rates are generally higher in high-altitude regions than in low-altitude areas, with a higher prevalence of chronic gastritis and greater severity of gastric mucosal lesions observed at high elevations (Ying et al., 2024; Requena et al., 2024; Lu et al., 2022). Therefore, investigating the pathogenesis of *H. pylori* gastritis under hypoxic conditions holds significant implications for maintaining gastrointestinal health in populations residing at high altitudes. Our previous clinical study showed that high-altitude hypoxia exacerbates the severity of chronic and active inflammation in the gastric mucosa of patients with *H. pylori* gastritis, as well as the oxidative stress and inflammatory response induced by *H. pylori* infection; however, the molecular mechanisms *in vivo* remain unclear (Li C. et al., 2024).

The gastric mucosa acts as the primary defense mechanism against *H*. *pylori* infection, with the impairment of its barrier function being pivotal in the pathogenesis of *H*. *pylori* gastritis (Oshima and Miwa, 2016). Oxidative stress and inflammatory responses induced by *H*. *pylori* are significant contributors to the breakdown of the gastric mucosal barrier and consequent mucosal injury (Suzuki and Hirai, 2024; Han et al., 2022). Nuclear factor erythroid two related factor 2 (Nrf2), as a key transcription factor in the body that regulates oxidative stress and inflammatory responses, is widely present in various tissues and cells, with Kelch-like ECH-associated protein 1 (Keap1) serving as its crucial regulatory protein (Suzuki et al., 2023; Baird and Yamamoto, 2020). Multiple studies have indicated that the Nrf2 signaling pathway plays a crucial role in maintaining gastric mucosal barrier function by alleviating oxidative stress, suppressing inflammatory responses, and preserving barrier integrity (Alomair et al., 2022; Duan et al., 2022; Gong et al., 2023; El Badawy et al., 2021; Ye et al., 2023). Furthermore hypobaric hypoxia induces oxidative stress and activates inflammatory responses through multiple pathways, thereby contributing to the onset and progression of various acute and chronic high-altitude diseases (Gaur et al., 2021), and the inhibition of the Nrf2 signaling pathway is one of the important mechanisms underlying this process (Duan H. et al.,. 2025; Han, et al., 2025; Han et al., 2025; Yu et al., 2025; Pan et al., 2023; Li X. Y. et al.,. 2024; Xiong et al., 2021). For example, Han et al. (Han et al., 2025) established a rat model of high-altitude cerebral edema by simulating a high-altitude hypoxic environment (6,000 m, 48 h) in a hypobaric chamber. The results showed that hypoxia induced the inactivation of the Nrf2 signaling pathway, which exacerbated oxidative stress and inflammatory responses, and ultimately aggravated brain injury. In addition, Yu et al. (2025) constructed a rat model of high-altitude pulmonary hypertension (HAPH) by simulating a high-altitude hypoxic environment (5,000 m, 4 weeks) using a hypobaric chamber. The results indicated that compared with the control group, the expression levels of Nrf2 and its downstream antioxidant proteins in the model group showed a significant downward trend, leading to increased oxidative stress. This may be one of the important mechanisms underlying hypoxia-induced pulmonary vascular remodeling and the occurrence and development of HAPH. The findings of Pan et al. (2023) also confirmed this observation. Furthermore, a study by Li X. Y. et al. (2024) revealed that under high-altitude hypoxic conditions, Keap1 was upregulated while Nrf2 was downregulated in the intestinal tissues of rats. This change reduced intestinal antioxidant capacity, exacerbated oxidative stress and inflammatory responses, and thereby promoted intestinal barrier damage, suggesting that the inhibition of the Nrf2 pathway may be a crucial mechanism for intestinal oxidative damage induced by high-altitude hypoxia.

This study established a mouse model of *H*. *pylori* infection under hypoxic conditions, systematically evaluated changes in the expression of gastric mucosal barrier-related proteins and key molecules of the Nrf2 signaling pathway, and detected oxidative stress and inflammatory markers in gastric tissue. It aims to elucidate the effects of high-altitude hypoxia on the gastric mucosal barrier and Nrf2 signaling pathway in *H*. *pylori*-infected mice, providing experimental evidence for deepening the understanding of the pathogenesis of *H*. *pylori* gastritis in high-altitude regions.

## 2 Materials and methods

### 2.1 Mice and reagents

Six-to eight-week-old male specific-pathogen-free C57BL/6 mice were purchased from Beijing Vital River Laboratory Animal Technology Co., Ltd. (Beijing, China) [certificate no. SCXK (Jing) 2021-0006]. Columbian blood agar medium was purchased from Kemajia Microbial Technology Co., Ltd. (Shanghai, China). A SPARKeasy Improved Tissue/Cell RNA Kit, SPARKscript II All-in-one RT SuperMix, and 2×SYBR Green qPCR Mix were purchased from Shandong Sparkjade Technology Co., Ltd. (Shandong, China). A bicinchoninic acid protein assay kit, malondialdehyde (MDA) assay kit, superoxide dismutase (SOD) activity assay kit, and glutathione peroxidase (GSH-Px) activity assay kit were purchased from Wuhan Abbkine Technology Co., Ltd. (Wuhan, China).

### 2.2 Bacterial strains and cultivation conditions

The Sydney standard strain of *H. pylori* was purchased from the Guangdong Microbial Strain Preservation Center. Frozen *H. pylori* strains were retrieved from an ultralow temperature freezer (−80 C), and a small portion of the bacterial culture was thawed and plated on Columbia blood agar. The plates were then incubated in a tri-gas incubator at 37 C with 5% O_2_, 10% CO_2_, and 85% N_2_ for 72 h. The second-generation *H. pylori* strains were subsequently cultured in brain-heart infusion (BHI), and their concentrations were adjusted to approximately 1 × 10^9^ colony forming units (CFU)/mL based on spectrophotometry-determined absorbance values at 600 nm.

### 2.3 Animal experiment

The animal experiments were approved by the Medical Ethics Committee of Qinghai University Affiliated Hospital (P-SL-2023-456) and performed in accordance with the recommendations of the National Laboratory Animal Care and Use Research Committee. The mice were kept in a regulated environment, with a 12-h light/dark cycle, and were provided with food and water available at all times. The animal room was maintained at a consistent temperature of 22 C–26 C and a relative humidity of 50%–60%. After 1 week of adaptive feeding, mice were randomly allocated to one of four experimental groups using a computer-generated random number sequence to ensure an equal probability of assignment: the control group (Con), the hypoxia group (H), the *H. pylori* infection group (Hp), and the combined *H. pylori* infection with hypoxia group (HpH). The Hp and HpH groups received 0.3 mL of *H. pylori* suspension in BHI *via* oral gavage every other day for a total of five doses, whereas the Con and H groups received 0.3 mL of normal saline on the same schedule. All mice were fasted for 12 h before gavage and remained fasted and water-deprived for 4 h post-gavage. Following the treatment period, mice in the Con and Hp groups were maintained in a control area (Xining, altitude 2,261 m) for 4 weeks, while those in the H and HpH groups were continuously housed in a hypobaric chamber simulating an altitude of 5,000 m for the same duration. The chamber was calibrated weekly using a precision barometer and oxygen analyzer to maintain a stable internal pressure of 52.9 kPa and oxygen partial pressure of 84.7 mmHg. In this study, due to the limited size of the research team and the overlap of key researchers responsible for animal grouping, intervention, sampling, and subsequent experimental operations, we were unable to strictly implement complete investigator blinding.

### 2.4 Sample collection

All mice underwent a 12-h fasting period before blood collection from the orbital venous plexus for complete blood count analysis under anesthesia. Subsequently, euthanasia was performed *via* cervical dislocation. The abdominal cavity was swiftly opened using sterile surgical instruments to extract the entire mouse stomach. An incision along the greater curvature exposed the gastric mucosa, allowing for washing of gastric contents with physiological saline. A segment from the middle portion of the lesser curvature was excised, fixed in 4% paraformaldehyde, embedded, and sectioned for histopathological examination. The remaining gastric tissue was placed in sterile cryogenic tubes, promptly frozen in liquid nitrogen, and stored at −80 C for molecular biological analysis.

### 2.5 Histopathological analysis

The gastric tissues underwent dehydration using alcohol, were then embedded in paraffin following a standard protocol, and finally cut to a thickness of 4 μm before being stained with haematoxylin and eosin (HE) for histopathology. Scoring of gastric epidermal damage were conducted using the following criteria: normal mucosa was assigned one point, damage to superficial mucosal cells received two points, involvement of glandular cells was given three points, and mucosal erosion, hemorrhage, or ulcer formation were allotted four points.

### 2.6 Immunohistochemical analysis

The paraffin sections were first dewaxed in water, then exposed to high-temperature antigen retrieval, followed by blocking with bovine serum albumin at room temperature. They were subsequently incubated with primary and secondary antibodies, visualized *via* DAB staining, counterstained with haematoxylin, dehydrated, and finally mounted. Five sections from each group were analyzed, with three discontinuous positive expression areas selected and photographed under ×400 magnification. ImageJ software was employed to calculate the percentage of positive area relative to the total selected area for semiquantitative analysis.

### 2.7 Reverse transcription-quantitative polymerase chain reaction (RT-qPCR)

Gastric tissue RNA was extracted through a column-based method, followed by reverse transcription of 1,000 ng of RNA into cDNA using a reverse transcription kit. Subsequently, RT-qPCR was carried out using SYBR Green PCR Mix on a ROCHE LightCycler 480 II System. The specific primers used are outlined in Table 1. The data were normalized with β-actin as a reference, and the relative mRNA expression was calculated using the 2^−ΔΔCp^ method.

**TABLE 1:** Information on primer sequences.

Gene	Forward primer	Reverse primer
β-actin	CATCCGTAAAGACCTCTATGCCAAC	ATGGAGCCACCGATCCACA
ZO-1	GTTGGAGCCAACTGTGTTTCTGTC	GTTCAATCCACGTTCACATTGCTTA
Occludin	AAGGTCCTGGTGTGAGCTGTGA	AGGGCTGCTGCAAAGATTGATTAG
TNF-α	CCCTCACACTCAGATCATCTTCT	GCTACGACGTGGGCTACAG
IL-1β	TCCAGGATGAGGACATGAGCAC	GAACGTCACACACCAGCAGGTTA
IL-6	GAGGATACCACTCCCAACAGACC	AAGTGCATCATCGTTGTTCAT

ZO-1, zonula occluden-1; TNF-α, tumor necrosis factor-α; IL, interleukin.

### 2.8 Western blot analysis

Gastric tissues were homogenized in pre-cooled lysis buffer (30 min), centrifuged (12,000 rpm, 5 min, 4 C), and supernatants were quantified *via* BCA assay. Proteins were separated *via* SDS-PAGE (140 V, 1 h) and electroblotted onto PVDF membranes (300 mA, 30 min). Membranes were blocked with protein-free rapid blocking buffer (20 min, RT), incubated with primary antibodies (overnight, 4 C), then HRP-conjugated secondary antibodies (1 h, RT). Signals were detected using an Amersham Imager 600 and analyzed with ImageJ software. Target protein expression was normalized to β-actin by gray value ratios. Primary antibodies used in this study were purchased from Wuhan Cloud-Clone Technology Co., Ltd. (Wuhan, China), including Keap1 antibody (1:1,000, PAL648Mu01), Nrf2 antibody (1:1,000, PAL947Mu01), heme oxygenase-1 (HO-1) antibody (1:800, PAA584Mu01), NAD(P) H quinone dehydrogenase 1 (NQO1) antibody (1:1,000, PAL969Mu01), B cell lymphoma-2 (Bcl-2) antibody (1:1,000, PAA778Mu01), Bcl-2 Associated X-protein (Bax) antibody (1:1,000, PAB343Mu01), Caspase-3 antibody (1:1,000, PAA626Mu01), and β-actin antibody (1:1,000, PAB340Mi01).

### 2.9 Evaluation of oxidative stress

Upon removal from the freezer, the frozen tissue was homogenized and centrifuged at 4 C and 12,000 rpm for 10 min to collect the supernatant. Following this, commercial kits were employed to analyze the MDA levels and the activity of SOD and GSH-Px in the gastric tissues.

### 2.10 Statistical analysis

Data analysis and graph generation were performed using SPSS 27.0 and GraphPad Prism 10.2.3. All data were subjected to the Shapiro-Wilk test for normality and Levene’s test for homogeneity of variance. For normally distributed data with equal variances, ANOVA followed by Tukey’s test was employed. For normally distributed data with unequal variances, ANOVA followed by Dunnett’s T3 test was used. Statistical significance was set at *p* < 0.05.

## 3 Results

### 3.1 Establishment of a mouse model of *H. pylori* infection under hypoxic conditions

Hemoglobin (Hb) and hematocrit (Hct) levels were assessed in mice post high-altitude hypoxic simulation to validate its efficacy. Results revealed a significant elevation in Hb and Hct levels in the H group compared to the Con group (all *p* < 0.001). Moreover, the HpH group displayed notably higher Hb and Hct levels than the Hp group (all *p* < 0.001), confirming the successful establishment of the hypoxia model (Table 2). Immunohistochemical staining was used to verify the colonization of *H. pylori* in the gastric mucosa of mice across all groups. The results showed that the gastric mucosa of mice in the Con and H groups were negative for *H. pylori* infection, while that of mice in the Hp and HpH groups were positive. These findings indicate the successful establishment of the *H. pylori* infection model.

**TABLE 2:** Hemoglobin (Hb) and hematocrit (Hct) values in each group of mice.

Groups	Hb (g/L)	Hct (%)
Con	127.75 ± 7.54	40.34 ± 3.45
H	168.63 ± 9.15^a^	53.03 ± 4.17^a^
Hp	129.63 ± 7.01	40.83 ± 3.89
HpH	171.38 ± 10.85^b^	54.15 ± 4.30^b^
*F*	59.43	28.83
*p*	<0.001	<0.001

Data were represented as mean ± SD (n = 8 mice/group) and analyzed using ANOVA, followed by Tukey’s test.

^a^ *p* < 0.05 VS., Con group.

^b^ *p* < 0.05 VS., Hp group.

### 3.2 The effect of hypoxia on gastric mucosal injury in mice with *H. pylori* infection

**FIGURE 1:**
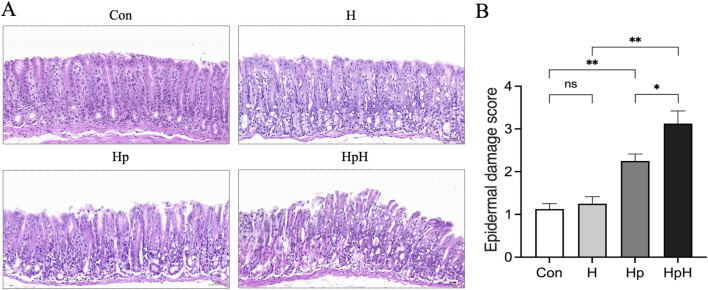
**(A)** HE staining of the gastric tissues (×200). Scale bar: 50 μm. **(B)** Epithelial damage scores of gastric tissue sections in each group. Data were represented as mean ± SEM (n = 8 mice/group) and analyzed using ANOVA followed by Tukey’s test. ^*^: *p* < 0.05, ^**^: *p* < 0.01, ns: not significant.

HE staining was utilized to examine the gastric mucosa pathology in mice across different groups (Figure 1A). The results revealed that the gastric mucosal epithelial layer in the Con group and H group exhibited an intact structure, regular cellular arrangement, and no obvious inflammatory cell infiltration. In contrast, the gastric mucosal epithelium in the Hp group showed necrosis and exfoliation, accompanied by inflammatory cell infiltration in the mucosal layer. Additionally, the HpH group exhibited mucosal erosion along with the aforementioned pathological alterations. The gastric epithelial damage scores analysis (Figure 1B) revealed no statistically significant difference between the Con and H groups (*p* = 0.970). However, the scores of the Hp group were significantly greater than those of the Con group (*p* = 0.002). Furthermore, the scores of the HpH group were notably greater than those of the H group (*p* < 0.001) and the Hp group (*p* = 0.020).

### 3.3 The effect of hypoxia on the expression of tight junction proteins in mice with *H. pylori* infection

**FIGURE 2:**
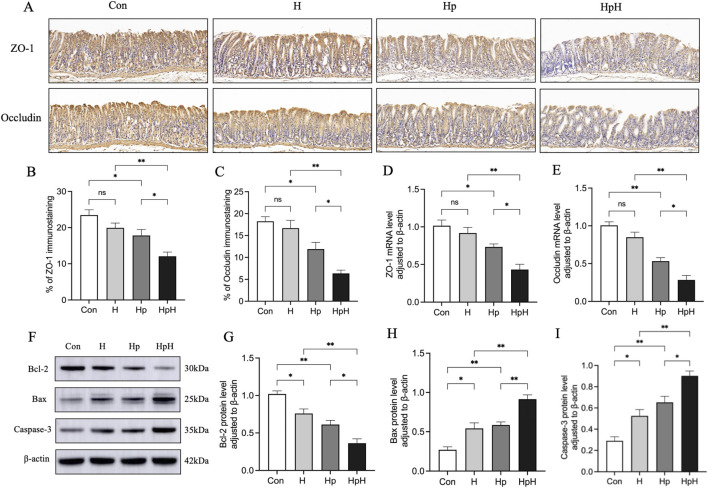
**(A–C)** Immunohistochemical staining and the positive area proportion of zonula occluden-1 (ZO-1) and Occludin in gastric (×200). Scale bar: 50 μm. Data were represented as mean ± SEM (n = 5 mice/group) and analyzed using ANOVA followed by Tukey’s test (ZO-1) or Dunnett’s T3 test (Occludin). **(D, E)** Relative mRNA expression of ZO-1 and Occludin in gastric. Data were represented as mean ± SEM (n = 6 mice/group) and analyzed using ANOVA followed by Tukey’s test. **(F–I)** Western blot detection of relative protein expression of B cell lymphoma-2 (Bcl-2), Bcl-2 Associated X-protein (Bax) and Caspase-3 in gastric. Data were represented as mean ± SEM (n = 4 mice/group) and analyzed using ANOVA followed by Tukey’s test. ^*^: *p* < 0.05, ^**^: *p* < 0.01.

RT-qPCR was employed to assess Zonula occluden-1 (ZO-1) and Occludin gene expression levels in gastric tissues of mice (Figures 2D,E). Results revealed no significant differences in ZO-1 (*p* = 0.733) and Occludin (*p* = 0.221) mRNA expression levels between the Con and H groups. The Hp group exhibited significantly decreased mRNA expression levels of ZO-1 (*p* = 0.034) and Occludin (*p* < 0.001) compared to the Con group. Furthermore, the HpH group displayed significantly lower mRNA expression levels of both ZO-1 and Occludin compared to the H group (all *p* < 0.001). Lastly, compared to the Hp group, the HpH group exhibited significantly reduced mRNA expression levels of ZO-1 (*p* = 0.021) and Occludin (*p* = 0.023).

Immunohistochemical staining was utilized to assess the protein expression levels of ZO-1 and Occludin in gastric tissues of mice (Figures 2A–C). The results revealed that, compared to the Con group, there were no significant differences in the protein expression levels of ZO-1 (*p* = 0.304) and Occludin (*p* = 0.969) in the H group. Conversely, the Hp group exhibited a significant decrease in the protein expression levels of ZO-1 (*p* = 0.033) and Occludin (*p* = 0.013) compared to the Con group. Moreover, the HpH group displayed a markedly reduced protein expression of both ZO-1 (*p* = 0.001) and Occludin (*p* < 0.001) compared to the H group. Additionally, in comparison to the Hp group, the HpH group also demonstrated a significant decrease in the protein expression levels of ZO-1 (*p* = 0.030) and Occludin (*p* = 0.022).

### 3.4 The effect of hypoxia on the expression of apoptosis-related proteins in mice with *H. pylori* infection

Protein expression levels of Bcl-2, Bax, and Caspase-3 in gastric tissues of mice were assessed using Western blot analysis (Figures 2F–I). The results indicated that, compared to the Con group, Bcl-2 expression was significantly reduced in the H group (*p* = 0.026), while Bax (*p* = 0.014) and Caspase-3 (*p* = 0.031) levels were significantly elevated. In the Hp group, Bcl-2 expression was significantly decreased (*p* = 0.001), with increased Bax (*p* = 0.005) and Caspase-3 (*p* = 0.002) levels. The HpH group displayed a significant decrease in Bcl-2 expression (*p* = 0.001) and significant increases in Bax (*p* = 0.001) and Caspase-3 (*p* = 0.001) expression compared to the H group. Furthermore, compared to the Hp group, the HpH group exhibited significantly lower Bcl-2 expression (*p* = 0.032) and significantly higher levels of Bax (*p* = 0.004) and Caspase-3 (*p* = 0.022) expression.

### 3.5 The effect of hypoxia on oxidative stress in mice with *H. pylori* infection

**FIGURE 3:**
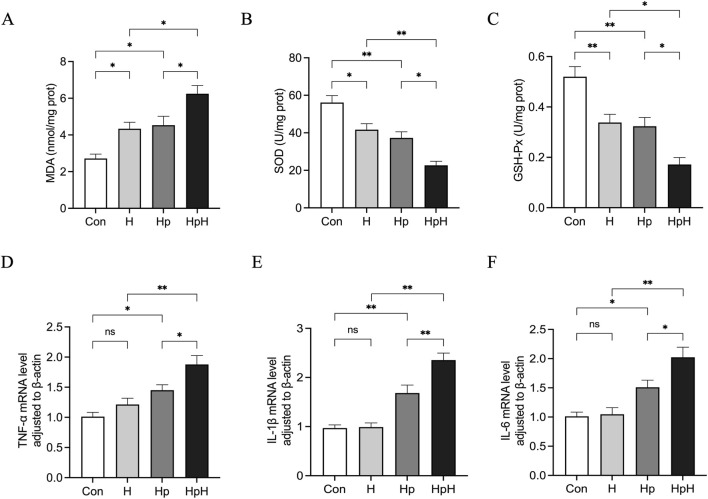
**(A–C)** The levels of malondialdehyde (MDA), superoxide dismutase (SOD), glutathione peroxidase (GSH-Px) in gastric. **(D–F)** Relative mRNA expression of tumor necrosis factor-α (TNF-α), Interleukin-1β (IL-1β), Interleukin-6 (IL-6) in gastric. Data were represented as mean ± SEM (n = 6 mice/group) and analyzed using ANOVA followed by Tukey’s test. ^*^: *p* < 0.05, ^**^: *p* < 0.01.

Biochemical analysis was conducted to assess the levels of MDA and the activities of SOD and GSH-Px in gastric tissues of mice (Figures 3A–C). The results revealed that the H group displayed significantly increased MDA levels (*p* = 0.039) and notably decreased SOD (*p* = 0.018) and GSH-Px (*p* = 0.006) activities compared to the Con group. The Hp group exhibited significantly elevated MDA levels (*p* = 0.019) and markedly reduced SOD (*p* = 0.002) and GSH-Px (*p* = 0.003) activities. Furthermore, the HpH group demonstrated significantly elevated MDA levels (*p* = 0.013) and significantly reduced SOD (*p* = 0.002) and GSH-Px (*p* = 0.012) activities compared to the H group. Lastly, compared to the Hp group, the HpH group showed significantly increased MDA levels (*p* = 0.028) and significantly decreased SOD (*p* = 0.017) and GSH-Px activities (*p* = 0.024).

### 3.6 The effect of hypoxia on inflammation in mice with *H. pylori* infection

RT-qPCR was used to assess TNF-α, IL-1β, and IL-6 gene expression in gastric tissues of mice (Figures 3D–F). The findings revealed no significant differences in the mRNA expression levels of TNF-α (*p* = 0.550), IL-1β (*p* = 0.991), or IL-6 (*p* = 0.997) between the Con and H groups. However, the Hp group exhibited notably higher mRNA expression of TNF-α (*p* = 0.039), IL-1β (*p* = 0.002), and IL-6 (*p* = 0.043) compared to the Con group. Furthermore, the HpH group demonstrated significantly increased mRNA expression of TNF-α (*p* = 0.001), IL-1β (*p* < 0.001), and IL-6 (*p* < 0.001) compared to the H group. In addition, mRNA levels of TNF-α (*p* = 0.042), IL-1β (*p* = 0.004), and IL-6 (*p* = 0.040) were significantly higher in the HpH group compared to the Hp group.

### 3.7 The effect of hypoxia on Keap1/Nrf2 pathway in mice with *H. pylori* infection

**FIGURE 4:**
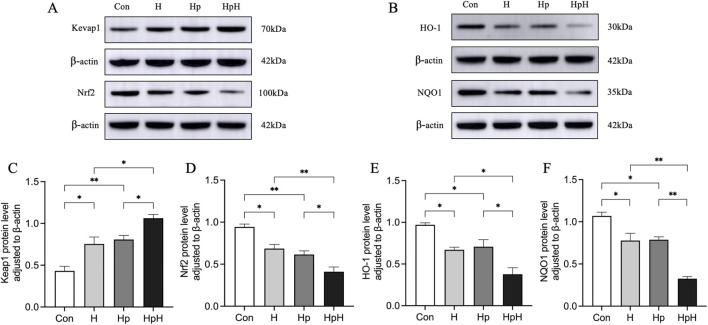
**(A–F)** Western blot detection of relative protein expression of Kelch-like ECH-associated protein 1 (Keap1), Nuclear factor erythroid two related factor 2 (Nrf2), heme oxygenase-1 (HO-1), NAD(P) H quinone dehydrogenase 1 (NQO1) in gastric. Data were represented as mean ± SEM (n = 4 mice/group) and analyzed using ANOVA followed by Tukey’s test. ^*^: *p* < 0.05, ^**^: *p* < 0.01.

Western blot was utilized to assess the protein expression levels of Keap1, Nrf2, HO-1, and NQO1 in gastric tissues (Figures 4A–F). The results indicated elevated Keap1 expression (*p* = 0.011) and reduced levels of Nrf2 (*p* = 0.010), HO-1 (*p* = 0.020), and NQO1 (*p* = 0.010) compared to the Con group. In the Hp group, Keap1 expression increased (*p* = 0.004), while Nrf2 (*p* = 0.002), HO-1 (*p* = 0.043), and NQO1 (*p* = 0.012) decreased. The HpH group showed increased Keap1 expression (*p* = 0.016) and decreased Nrf2 (*p* = 0.007), HO-1 (*p* = 0.023), and NQO1 (*p* < 0.001) compared to the H group. Additionally, the HpH group exhibited significantly higher Keap1 expression (*p* = 0.041) and lower Nrf2 (*p* = 0.042), HO-1 (*p* = 0.011), and NQO1 (*p* < 0.001) levels compared to the Hp group.

**FIGURE 5:**
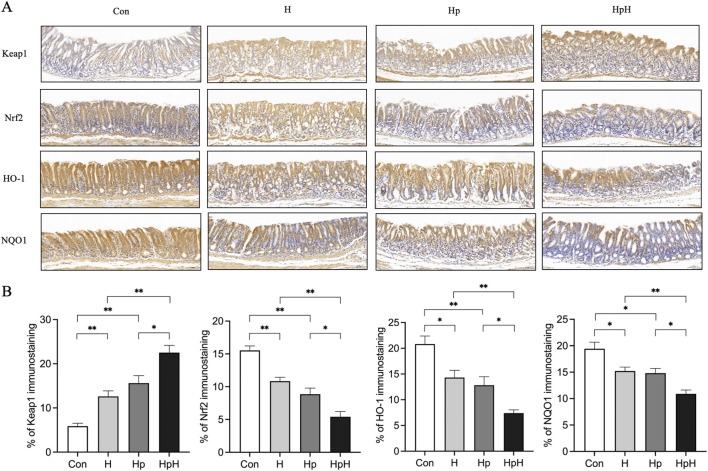
**(A, B)** Immunohistochemical staining and the positive area proportion of Kelch-like ECH-associated protein 1 (Keap1), Nuclear factor erythroid two related factor 2 (Nrf2), heme oxygenase-1 (HO-1), NAD(P) H quinone dehydrogenase 1 (NQO1) in gastric (×200). Scale bar: 50 μm. Data were represented as mean ± SEM (n = 5 mice/group) and analyzed using ANOVA followed by Tukey’s test (Nrf2) or Dunnett’s T3 test (Keap1, HO-1, and NQO1). ^*^: *p* < 0.05, ^**^: *p* < 0.01.

Immunohistochemical analysis (Figures 5A,B) of gastric tissue revealed that the H group displayed elevated Keap1 expression (*p* = 0.001) compared to the Con group, while Nrf2 (*p* < 0.001), HO-1 (*p* = 0.025), and NQO1 (*p* < 0.042) levels were reduced. In the Hp group, Keap1 expression increased (*p* < 0.001), whereas Nrf2 (*p* < 0.001), HO-1 (*p* = 0.008), and NQO1 (*p* = 0.033) levels decreased. The HpH group exhibited significantly higher Keap1 expression (*p* < 0.001) and significantly lower Nrf2 (*p* < 0.001), HO-1 (*p* = 0.002), and NQO1 (*p* = 0.002) levels compared to the H group. In comparison with the Hp group, the HpH group showed increased Keap1 expression (*p* = 0.037) and decreased Nrf2 (*p* = 0.012), HO-1 (*p* = 0.036), and NQO1 (*p* = 0.013) levels.

## 4 Discussion

Compared with our previous clinical observation (Li C. et al.,. 2024), this study established a mouse model of *H. pylori* infection under hypoxic conditions to reveal the impact of high-altitude hypoxia on *H. pylori*-induced gastric mucosal injury and its underlying mechanisms.

Regarding pathological damage, HE staining revealed that the hypoxic environment alone had minimal impact on gastric mucosal structure and did not cause significant pathological injury. The Hp group exhibited marked pathological alterations in the gastric mucosa, including epithelial cell necrosis and exfoliation, and inflammatory cell infiltration, consistent with previous studies (Sayed et al., 2020). Furthermore, the HpH group exhibited more severe pathological alterations than both the Hp and H groups, accompanied by mucosal erosion and significantly elevated gastric epidermal damage scores. This indicates that hypoxia significantly exacerbates gastric mucosal damage in *H*. *pylori*-infected mice.

The gastric mucosal barrier serves as the body’s first line of defense against *H. pylori* infection, and its integrity is crucial for maintaining gastric homeostasis. Among its components, the epithelial barrier formed by epithelial cells connected *via* tight junctions is the most important structural element for sustaining the function of the gastric mucosal barrier (Ye et al., 2023; Zhao et al., 2024). ZO-1 and Occludin are involved in cytoskeleton formation, so they are important structures of tight junctions (Zuo et al., 2020). Previous research has shown that *H. pylori* infection diminishes ZO-1 and Occludin expression in the gastric mucosa, inducing apoptosis and facilitating *H. pylori* colonization, worsening gastric mucosal damage (Teng et al., 2020; Lim et al., 2023). Our study showed that compared with the Con group, the expression levels of ZO-1 and Occludin in gastric tissue of the H group showed no significant changes, while those in the Hp group were significantly decreased. Meanwhile, compared with the Con group, both the H and Hp groups exhibited downregulation of the anti-apoptotic protein Bcl-2 and upregulation of the pro-apoptotic proteins Bax and Caspase-3. These findings indicate that although hypoxia alone has no significant effect on the expression of tight junction proteins in gastric tissue, it can induce changes in the expression of apoptosis-related proteins. In contrast, *H. pylori* infection can both inhibit the expression of tight junction proteins in gastric tissue and promote abnormal expression of apoptosis-related proteins. Furthermore, the expression levels of ZO-1, Occludin, and Bcl-2 in gastric tissue of the HpH group were further reduced compared with those in the H group and Hp group, while the expression levels of Bax and Caspase-3 were further increased. The above experimental results suggest that high-altitude hypoxia may significantly exacerbate the destructive effects of *H. pylori* infection on the gastric mucosal barrier through certain mechanisms, leading to more severe gastric mucosal damage.

Oxidative stress and inflammatory responses are key mechanisms by which *H. pylori* infection disrupts the gastric mucosal barrier and causes mucosal damage (Suzuki and Hirai, 2024). The results of this study showed that compared with the Con group, the gastric tissue MDA levels in the H and Hp groups were significantly increased, while the activities of SOD and GSH-Px were significantly decreased, suggesting that either hypoxia alone or *H. pylori* infection can induce oxidative stress in the gastric mucosa. Previous studies have shown that *H. pylori* can increase the production of reactive oxygen species (ROS) through multiple mechanisms, such as activating inflammatory cells like neutrophils, impairing glutathione metabolism in gastric epithelial cells, and directly affecting mitochondrial function (Salvatori et al., 2023; Liu et al., 2024; Sah et al., 2023). Hypoxia itself can also cause mitochondrial dysfunction and increased ROS production (Pena et al., 2022; Mrakic-Sposta et al., 2021; Mrakic-Sposta et al., 2022), which is consistent with our findings. Furthermore, this study found that compared with the H and Hp groups, the HpH group exhibited further elevated MDA levels in gastric tissue alongside additional reductions in SOD and GSH-Px activity, indicating a synergistic effect between hypoxia and Hp infection in inducing gastric mucosal oxidative stress.

Oxidative stress and inflammatory responses are closely intertwined, forming a self-reinforcing vicious cycle where each exacerbates the other (Jomova et al., 2023; Duan Y. et al.,. 2025; Kim et al., 2018). Our findings revealed that the expression levels of pro-inflammatory factors TNF-α, IL-1β, and IL-6 in the gastric tissue of the Hp group were upregulated. This aligns with the mechanism whereby *H. pylori* infection activates innate and adaptive immune responses, leading to the release of substantial inflammatory mediators (Yang and Hu, 2022). Interestingly, the expression levels of the aforementioned pro-inflammatory factors in the H group were not significantly different from those in the Con group. However, the expression levels of TNF-α, IL-1β, and IL-6 in the HpH group were significantly higher than those in the H and Hp groups. This suggests that the oxidative stress induced synergistically by hypoxia and *H. pylori* may serve as a potent “second signal”, significantly amplifying downstream inflammatory responses. Excessive inflammatory responses not only cause direct tissue damage, but also recruit additional inflammatory cells. While exerting their immune functions, these cells produce increased levels of ROS, which further exacerbate oxidative stress. This cascade ultimately leads to the most severe gastric mucosal damage observed in the HpH group.

HO-1 and NQO1 are classic downstream target genes of the Nrf2 signaling pathway (O'Rourke et al., 2024; Ross and Siegel, 2021). Previous studies have demonstrated that *H. pylori* infection can suppress the expression of Nrf2 and its downstream genes, weakening the antioxidant defense capacity of the gastric mucosa and promoting inflammatory responses, thereby leading to gastric mucosal damage (Zhang et al., 2022; Kim et al., 2022; Wang et al., 2025). The results of this study showed that compared with the Con group, the expression levels of Keap1 in gastric tissues were elevated in both the H and Hp groups, while the levels of Nrf2 and its downstream target genes HO-1 and NQO1 were reduced. These findings indicate that both hypoxia alone and *H. pylori* infection can inhibit the activity of the Nrf2 signaling pathway. Furthermore, we found that compared with the H and Hp groups, the expression levels of Keap1 in gastric tissue were further elevated in the HpH group, while the levels of Nrf2, HO-1, and NQO1 were further reduced. This suggests that hypoxia synergistically suppresses the Nrf2 signaling pathway with *H. pylori* infection, and this cooperative inhibition of the core defense pathway may represent the molecular mechanism underlying the most severe oxidative stress, inflammatory response, barrier disruption, and gastric mucosal damage observed in the HpH group. Notably, the activity of Nrf2 is subject to interactive regulation by multiple signaling pathways, particularly through intimate crosstalk with hypoxia-inducible factor-1α (HIF-1α) and the nuclear factor-kappa B (NF-κB) pathway, a central mediator of inflammation. Under hypoxic conditions, cells rapidly activate the HIF-1α pathway to adapt to hypoxic stress (Lee et al., 2020), and a complex positive or negative regulatory relationship exists between HIF-1α and Nrf2 (Bae et al., 2024). Meanwhile, *H*. *pylori* infection activates NF-κB pathway *via* its virulence factors (Maubach et al., 2022). NF-κB and Nrf2 typically exhibit a mutual antagonistic interaction: NF-κB activation suppresses Nrf2 signaling, whereas Nrf2 activation inhibits excessive NF-κB-mediated inflammatory responses (Saha et al., 2020). The strongest suppression of the Nrf2 pathway observed in the HpH group in this study may result from a synergistic effect between hypoxia-induced HIF-1α activation and *H*. *pylori* infection-mediated NF-κB activation, jointly regulating the Nrf2 signaling pathway. Future research should further explore the cross-regulatory mechanisms among these three pathways at the molecular level.

### 4.1 Limitations

Despite the significant findings of this study, several limitations should be acknowledged. First, we did not perform functional manipulation of the Nrf2 signaling pathway, such as through genetic knockout or pharmacological activation, which would have provided more direct evidence for its causal role in mediating hypoxia-exacerbated *H. pylori*-induced gastric mucosal injury. Second, the sample size for some experimental techniques was relatively small, which may limit statistical power, and this study only used male mice, failing to exclude the potential impact of gender differences on *H. pylori*-pathogenesis and hypoxic responses. Third, the study was conducted at a single time point after intervention, which may not capture dynamic changes in pathological and molecular responses over time. Fourth, only one standard strain of *H. pylori* was used, which may not fully represent the diversity of clinical isolates and their varying pathogenic potentials. Finally, we did not perform precise quantification of bacterial colonization in gastric tissues, which could have provided more detailed correlation between bacterial load and mucosal damage severity. Future studies should address these limitations to validate and extend our findings.

## 5 Conclusion

In summary, this study demonstrates that high-altitude hypoxia significantly exacerbates oxidative stress and inflammatory responses induced by *H. pylori* infection, reduces the expression of tight junction proteins in gastric tissues and triggers changes in the expression of apoptosis-related proteins, thereby worsening the disruption of gastric mucosal barrier function caused by *H. pylori* infection and ultimately leading to more severe gastric mucosal injury. The underlying mechanism may be associated with the inhibition of the Nrf2 signaling pathway in gastric tissues of *H. pylori*-infected mice. This study provides experimental evidence for studying the pathogenesis of *H. pylori* gastritis in high-altitude regions, but direct therapeutic implications require further validation *via* Nrf2-targeted functional experiments.

## Data Availability

The original contributions presented in the study are included in the article/supplementary material, further inquiries can be directed to the corresponding author.
